# Occult insulinoma with treatment refractory, severe hypoglycaemia in multiple endocrine neoplasia type 1 syndrome; difficulties faced during diagnosis, localization and management; a case report

**DOI:** 10.1186/s12902-022-00985-w

**Published:** 2022-03-16

**Authors:** Rasika Ranaweerage, Shehan Perera, Harsha Sathischandra

**Affiliations:** Registrar in General Medicine, National Hospital of Sri Lanka, Ward 45/46, Colombo, Sri Lanka

**Keywords:** Multiple endocrine Neoplasia type 1, Collagenomas, Insulinoma, Selective arterial calcium stimulation test, Refractory hypoglycaemia

## Abstract

**Background:**

Multiple endocrine neoplasia type 1 (MEN 1) syndrome is a rare, complex genetic disorder characterized by increased predisposition to tumorigenesis in multiple endocrine and non-endocrine tissues. Diagnosis and management of MEN 1 syndrome is challenging due to its vast heterogeneity in clinical presentation.

**Case presentation:**

A 23-year-old female, previously diagnosed with Polycystic Ovarian Syndrome (PCOS) and pituitary microprolactinoma presented with drowsiness,confusion and profuse sweating developing over a period of one day. It was preceded by fluctuating, hallucinatory behavior for two weeks duration. There was recent increase in appetite with significant weight gain. There was no fever, seizures or symptoms suggestive of meningism. Her Body mass index(BMI) was 32 kg/m^2^.She had signs of hyperandrogenism. Multiple cutaneous collagenomas were noted on anterior chest and abdominal wall. Her Glasgow Coma Scale was 9/15. Pupils were sluggishly reactive to light. Tendon reflexes were exaggerated with up going planter reflexes. Moderate hepatomegaly was present. Rest of the clinical examination was normal. Laboratory evaluation confirmed endogenous hyperinsulinaemic hypoglycaemia suggestive of an insulinoma. Hypercalcemia with elevated parathyroid hormone level suggested a parathyroid adenoma. Presence of insulinoma, primary hyperparathyroidism and pituitary microadenoma, in 3^rd^ decade of life with characteristic cutaneous tumours was suggestive of a clinical diagnosis of MEN 1 syndrome. Recurrent, severe hypoglycaemia complicated with hypoglycaemic encephalopathy refractory to continuous, parenteral glucose supplementation and optimal pharmacotherapy complicated the clinical course. Insulinoma was localized with selective arterial calcium stimulation test. Distal pancreatectomy and four gland parathyroidectomy was performed leading to resolution of symptoms.

**Conclusions:**

Renal calculi or characteristic cutaneous lesions might be the only forewarning clinical manifestations of an undiagnosed MEN 1 syndrome impending a life-threatening presentation. Comprehensive management of MEN 1 syndrome requires multi-disciplinary approach with advanced imaging modalities, advanced surgical procedures and long-term follow up due to its heterogeneous presentation and the varying severity depending on the disease phenotype.

## Background

Multiple endocrine neoplasia type 1 (MEN 1) is a rare, complex genetic disorder characterized by increased predisposition to occurrence of tumors in multiple endocrine and non-endocrine tissues including skin. MEN 1 syndrome is defined clinically as the occurrence of two or more MEN 1 associated endocrine tumours or the occurrence of one MEN 1 syndrome associated endocrine tumour in a patient with family history of MEN 1 syndrome. Diagnosis and management of MEN 1 syndrome is a challenging task due to its heterogeneity in clinical presentation and requires a multidisciplinary approach.

Here, we discuss a case of a MEN 1 syndrome with insulinoma, parathyroid adenoma and pituitary microprolactinoma, with emphasis on the management of treatment refractory, severe hypoglycaemia and the challenges faced during diagnosis, localization and treatment of insulinoma.

## Case presentation

A 23-year-old female, previously diagnosed with Polycystic Ovarian Syndrome( PCOS) and pituitary macroprolactinoma presented with drowsiness, confusion and profuse sweating developing over a period of one day. Parents revealed that she was having a hallucinatory behavior that is both auditory and visual, in the preceding two weeks. She did not have fever, seizures or symptoms suggestive of meningism. There was recent increase in appetite with significant weight gain. Parents could not recall any improvement of symptoms following the meals. She neither smoked or consumed alcohol and did not consume any psychoactive substances. She did not travel recently.

At the age of 16 years, she was evaluated for hirsutism and oligomenorrhoea by a gynaecologist and was found to have bilateral, multiple ovarian cysts on ultrasound scan. She was diagnosed with PCOS and was advised on lifestyle modification including weight reduction. Serum Prolactin, Thyroid Stimulating Hormone (TSH) and total Testosterone levels were within normal range at that time. Four years later, she was re-evaluated by an endocrinologist for persistent oligomenorrhoea and hirsutism. She was diagnosed to have a pituitary microprolactinoma (7 mm × 5 mm) with a serum Prolactin level of 347 ng/mL( 4.04–15.2 ng/mL) while rest of her pituitary hormones, total Testosterone and Dehydroepiandrosterone (DHEA) levels were within normal range. Bilateral polycystic ovaries were noted during the repeat ultrasound scan of abdomen and pelvis. An asymptomatic, non-obstructing left side renal calculus was noted during this scan by the radiologist, but it was not further evaluated. She was started on Cabergoline 0.5 mg weekly but was lost to follow up thereafter. There was no family history of established familial multiple endocrine disorders but her paternal grandmother and aunt had a history of recurrent renal calculi. She was the first offspring of a non-consanguineous marriage.

**Fig. 1:**
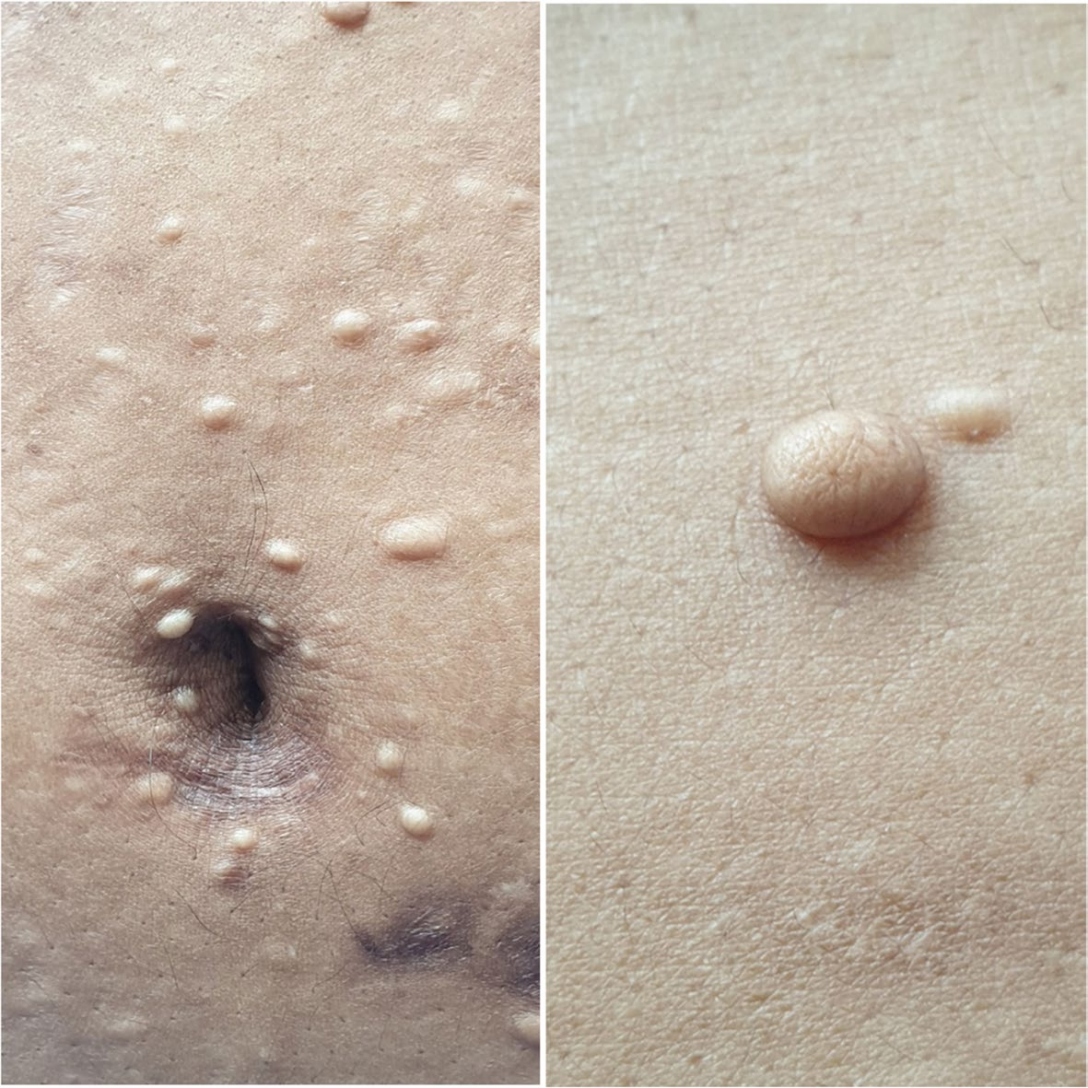
Multiple cutaneous collagenomas

Clinical examination revealed an obese lady with a Body mass index(BMI) of 32 kg/m^2^. She had signs of hyperandrogenism i.e.; hirsutism and acne vulgaris. There were multiple, skin coloured, dome shaped, smooth surfaced papules of 3-5 mm involving the anterior chest and abdominal wall suggestive of collagenomas which was later confirmed histologically as well (Fig. 1). Her Glasgow Coma Scale (GCS) score was 9/15. Patient was sweating profusely. Pulse rate was 110 beats per minute. Blood pressure was 120/80 mmHg. Neurological examination revealed sluggishly reactive pupils. Fundal examination was normal. The tendon reflexes were exaggerated with up going planter reflexes. Abdominal examination revealed a nontender, smooth surfaced, firm, moderate hepatomegaly. Rest of the clinical examination was normal.

Her Random plasma glucose level was 23.3 mg/dL on admission. She was treated with intravenous 20% dextrose boluses followed continuous infusion. But the patient continued to develop recurrent, severe hypoglycaemic episodes. Due to the prolonged reduced conscious level during the episodes of hypoglycaemia, elective intubation was carried out to protect the airway and to prevent aspiration.

Initial laboratory evaluation including full blood count and inflammatory markers were within normal parameters. Non-contrast Computed Tomography (CT) of brain was normal. Cerebrospinal fluid (CSF) analysis was normal except for very low sugar levels. Serum insulin level was 300 mU/L (fasting level < 25 mU/L) with a c-peptide level of 15.63 ng/mL (fasting level 0.78–1.89 ng/mL) which was drawn during an episode of confirmed severe hypoglycaemia. Toxicology screening did not reveal presence of oral hypoglycaemic agents. Her serum fasting gastrin level 70 pg/mL. Prolactin was 11,716 mU/L (300 – 500 mU/L). Serum Calcium (corrected to an albumin level of 3.2 g/dL) was 16.1 mg/dL(8.6–10.3 mg/dL) and serum phosphate level was 1.3 mg/dL(3–4.5 mg/dL). Intact parathyroid hormone (PTH) level was 329.7 pg/mL (18.4–80 pg/mL). 24-h urinary calcium excretion was 828.82 mg/dL (100 -300 mg/24 h). The findings were suggestive of endogenous hyperinsulinaemic hypoglycaemia and primary hyperparathyroidism. Thyroid stimulating hormone(TSH), Thyroxine (fT4), Cortisol, Growth hormone and Follicular stimulating hormone (FSH), Luteinizing hormone (LH), total Testosterone and DHEA levels were within the respective normal range.

Our patient was having recurrent, endogenous hyperinsulinaemic hypoglycaemia and primary hyperparathyroidism with multiple cutaneous collagenomas in the background of pituitary microadenoma and family history of recurrent nephrolithiasis. A unifying clinical diagnosis of MEN 1 syndrome appeared to be the likely explanation for current presentation.

The patient was extubated following a protracted recovery of conscious level with few residual neurological sequalae. But she continued to have frequent severe hypoglycaemic episodes even while on continuous intravenous 20% dextrose 100 mL/h infusions, Diazoxide 100 mg 8 hourly, Octreotide 100 µg 6 hourly, Growth hormone 2 mg daily and frequent high glycaemic index carbohydrate meals. Continuous 20% dextrose infusion with intermittent 50% dextrose boluses and hourly capillary blood glucose monitoring was required to maintain normoglycaemia. Diazoxide and Growth hormone were discontinued after one month due to poor response. Octreotide was continued considering its inhibitory action on pancreatic insulin secretion. None of these pharmacological treatments were able to completely ameliorate the hypoglycaemic episodes. Hypercalcemia was managed with volume expansion, a single dose of Zoledronic acid 4 mg and Cinacalcet 30 mg daily. Her hospital stay was further complicated with numerous episodes of ventilator associated pneumonia, infected vascular accesses and recurrent urinary tract infections. Repeated cultures isolated both bacterial and fungal organisms which required prolonged courses of systemic antibiotics and antifungal therapy.

**Fig. 2:**
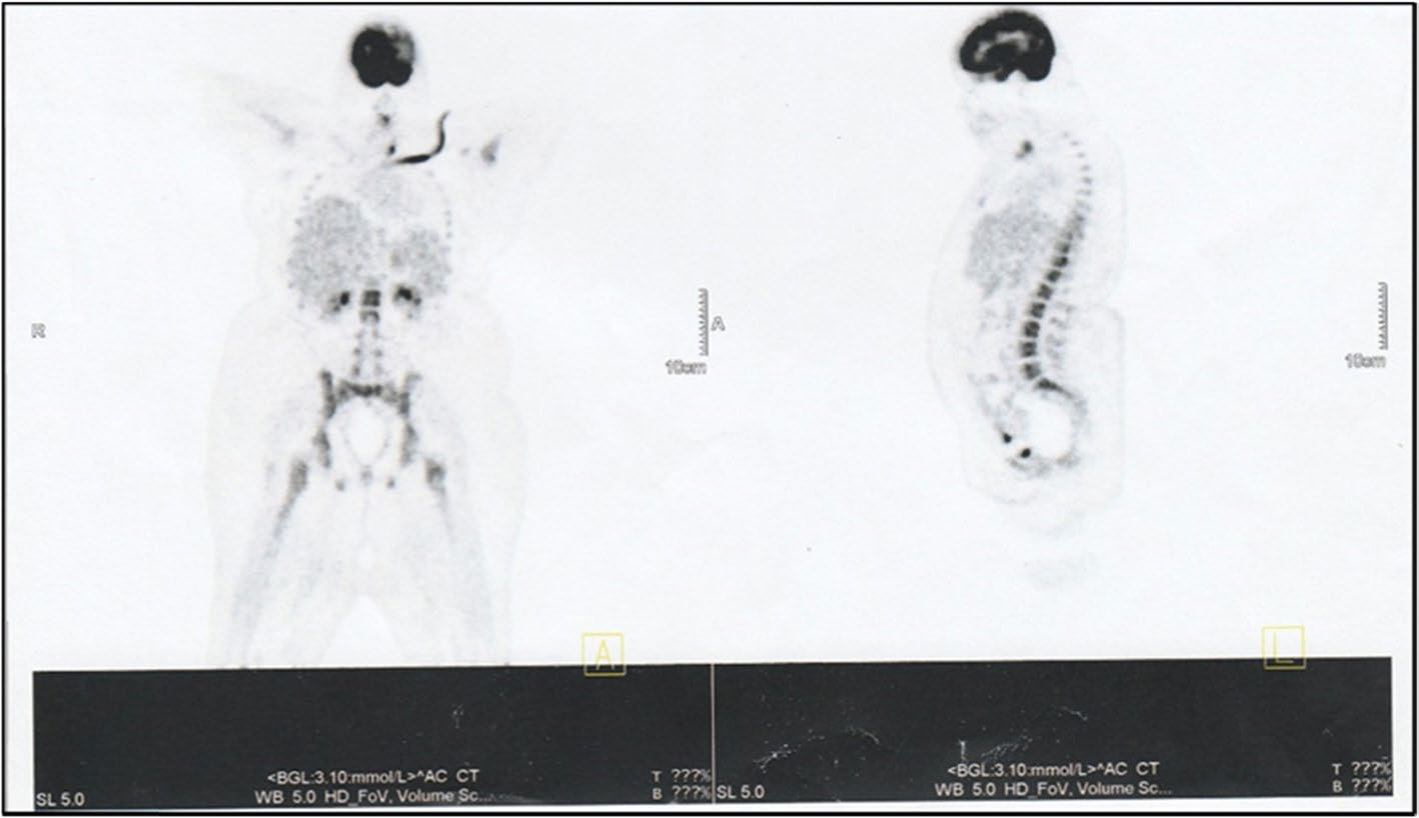
whole body PET/CT scan

Extensive radiological imaging was done to localize the tumours. Contrast enhanced Computed Tomography (CECT) scan of abdomen and pelvis revealed three focal lesions in liver segments II, IV and VII with enhancement in arterial phase and washout in the venous phase, suggestive of hypervascular lesions. Pancreatic imaging was normal. There was no evidence of renal calculi or suprarenal masses. A follow up Magnetic resonance imaging (MRI) of Abdomen reported that the focal liver lesions are more in favour of benign nature i.e. two focal lesions in segments II and IV are likely to be haemangiomas while the lesion is segment VII is suggestive of Focal nodular hyperplasia. It was noted that the pancreas is atrophic without presence of mass lesions or duct dilatation. Due to diagnostic uncertainty, a whole body fluorodeoxyglucose Positron Emission Tomography—Computed Tomography (FDG PET/CT) scan was performed (Fig. 2). It did not show any metabolically active lesions in liver or pancreas.

**Fig. 3:**
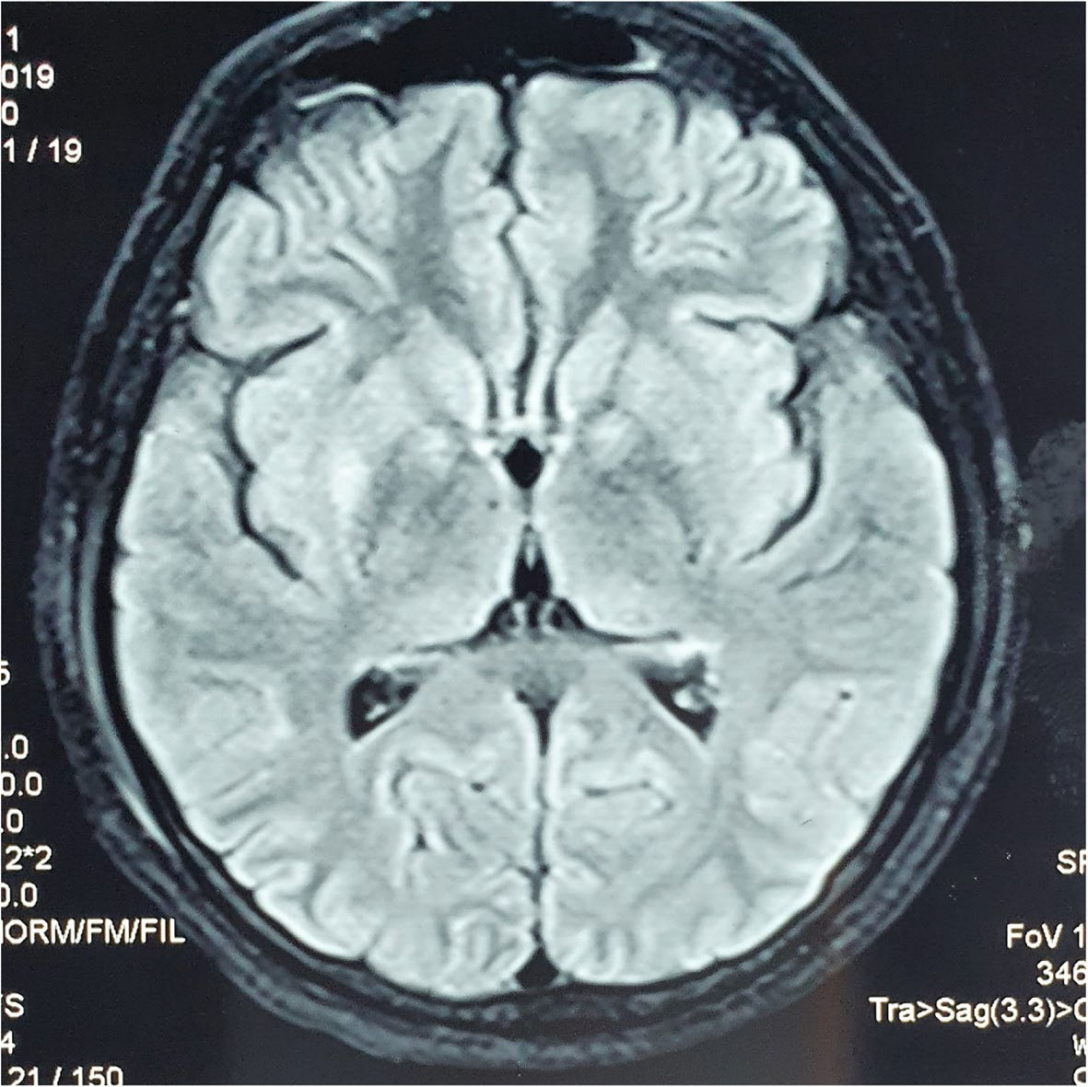
MRI Brain T2 FLAIR- Hypoglycaemic encephalopathy

MRI brain and neck revealed T2/FLAIR (Fluid-attenuated inversion recovery) hyperintensity with diffusion restriction in bilateral globus pallidi, left parieto-occipital cortex and bilateral temporal lobe cortices medially, which was in favour of hypoglycaemic encephalopathy (Fig. 3). The pituitary gland was normal in size with a hypointense lesion in left lobe that was consistent with her history of pituitary microadenoma.

During the localization of suspected the parathyroid adenoma, soft tissues in neck region was not visualized properly either with ultrasound imaging, CECT or MRI imaging which was attributed to the obese body habitus of the patient. Hence further evaluation with nuclear imaging was suggested but the necessary nuclear isotope imaging facility to localize parathyroid adenoma was not available temporarily in our institute during that time.

Since none of the imaging modalities were able to localize the insulinoma, a selective arterial calcium stimulation test (SACST) was performed. It was able to localize the insulinoma to the distal body of the pancreas. Following successful localization of insulinoma, feasibility of surgical resection was discussed in the multidisciplinary team meeting (MDT). MDT concluded that the patient was having high risk of surgery related mortality due to ongoing sepsis and resistant hypoglycaemia. Percutaneous chemoembolization appeared to be a favorable treatment option at that time. Chemoembolization was performed following the successful identification of vascular supply to the insulinoma with intraprocedural angiography. The following day, patient developed severe acute pancreatitis complicated with acute respiratory distress syndrome and required intensive care. Chemoembolization appeared to be unsuccessful since the patient continued to have hypoglycaemic episodes. MDT decided that the patient requires an open surgery to resect distal pancreatic insulinoma. Following extensive preoperative optimization and planning, an open distal pancreatectomy with splenectomy was performed. Histological assessment revealed a well differentiated pancreatic islet cell tumour. Following surgery, hypoglycaemia improved dramatically with normalization of blood glucose, serum insulin and c-peptide levels. Three weeks after the first surgery, patient underwent standard four gland exploration of parathyroid glands which revealed two large parathyroid glands on left side. Four gland parathyroidectomy was performed followed by implantation. Later they were histologically confirmed as parathyroid adenomas.

Following satisfactory clinical and biochemical recovery, patient was discharged home after 6 months from her admission. Prior to discharge, she underwent a formal cognitive assessment which showed mild deficits in executive functioning, language, memory and processing speed domains. She was arranged follow up with psychiatric services and occupational therapy. Follow-up serum insulin, c-peptide, intact PTH, and calcium levels were found to be within the normal range. Two months from discharge, she presented with brief episode of right side, tonic clonic, focal seizure with preserved awareness. Blood glucose, serum insulin, c -peptide, intact PTH, and calcium levels were found to be normal. MRI brain was repeated which revealed an area of gliosis in left side temporal lobe. The area of gliosis corresponded with the MRI brain that was performed during the previous admission, which demonstrated the typical features of hypoglycaemic encephalopathy. She was evaluated by a neurologist and was diagnosed with a remote symptomatic seizure. She was started on antiepileptic medication to prevent recurrent seizures and progressive brain injury. Currently she is being follow-up regularly and continues to be on Cabergoline for prolactin secreting pituitary microadenoma. Biochemical screening of first-degree family members was also done which was found to be negative, but we were not able to screen the two relatives who had history of recurrent nephrolithiasis. Unfortunately, facilities to carry out the genetic analysis is not available locally. The endocrine team is currently in discussion with a research centre in United Kingdom to arrange the genetic studies for the patient and the family members.

## Discussion and conclusions

Multiple Endocrine Neoplasia type 1 (MEN 1) syndrome is a rare, autosomal dominantly inherited genetic disorder. It was first described by Dr. Paul Wermer in 1954. In more than 80% of the cases, it is caused by inactivating mutation on MEN 1 gene located on chromosome 11q13. MEN 1 appears to be a tumour suppressor gene but precise mechanisms of tumorigenesis are not established yet. Both sexes are equally affected with a prevalence of 2:100,000 [1]. Presence of mutated MEN 1 gene results in increased predisposition to hyperplasia of endocrine glands and non-endocrine organs. Among the endocrine tumours, parathyroid adenoma (95%), pancreatic islet cell tumours (gastrinoma, insulinoma) (40%), pituitary adenoma (30%) and adrenal cortical adenoma, carcinoid tumours are frequently found. Ovarian tumours, lipomas, collagenomas and angiofibromas are noted to be manifested as non-endocrine tumours.

MEN 1 syndrome is defined clinically as the occurrence of two or more MEN 1 associated endocrine tumours or the occurrence of one MEN 1 syndrome associated endocrine tumour in a patient with family history of MEN 1 syndrome. Genetic testing for MEN 1 mutation is available for clinical use and should be considered on individual basis. But presymptomatic genetic diagnosis has not shown any clear benefits in preventing of morbidity and mortality in individuals with risk of developing MEN 1 syndrome [2]. In our patient, presence of endogenous hyperinsulinaemic hypoglycaemia was suggestive of an insulinoma. Coexistence of primary hyperparathyroidism, pituitary microadenoma and characteristic cutaneous tumours in a patient diagnosed with an insulinoma in her 3^rd^ decade of life led to a diagnosis of MEN 1 syndrome. Insulinoma in MEN 1 syndrome typically presents in the second to fourth decade of life as opposed to sporadic insulinoma, which usually occurs in individuals older than 40 years [2]. Our patient did not have any family members diagnosed with MEN 1 syndrome but had two paternal relatives with history of recurrent renal calculi who were not extensively evaluated.

The main obstacle we encountered during the evaluation and management of our patient was that she continued to develop recurrent, level 3 hypoglycaemia, that was refractory to both parenteral and enteral glucose replacement with pharmacotherapy including Diazoxide, Octreotide and Growth hormone. Patient was mostly managed in a high dependency unit from admission to the time of surgery, to minimize the episodes of unobserved neuroglycopenia. Despite of our best efforts, she had multiple episodes of break through hypoglycaemia that resulted in hypoglycaemic encephalopathy and chronic brain injury with mild residual cognitive deficits. Inability to maintain normoglycaemia ultimately resulted in delaying of diagnostic imaging, localization of insulinoma and therapeutic interventions. It was suspected that the aggressive phenotypical presentation of the patient could be due to, either the presence of a malignant insulinoma with metastasis, the multifocal nature of insulinoma or due to the presence of certain genetic polymorphisms associated with aggressive phenotypes [3]. The genotype—phenotype relationship in MEN 1 syndrome is yet to be proven due to the complex heterogeneity of MEN 1 gene mutations [3]. But occurrence of certain additional genetical polymorphisms in the presence of MEN 1 gene mutations have proven to result in aggressive disease phenotypes [4].

Despite of prompt clinical diagnosis, localization of insulinoma was a difficult task which ultimately required a SACST with venous sampling since none of the conventional imaging modalities were able to demonstrate the insulinoma. SACST confirmed the diagnosis of insulinoma while localizing the tumour to distal body of the pancreas and identified the feeding vessels thus providing us with valuable information in planning and execution of resection of tumour. According to a study carried out by Mayo clinic over a couple of decades, the success rate of identifying insulinoma with CT and transabdominal ultrasonography was approximately 75% [5]. Some studies found that transabdominal ultrasonography to have a lesser sensitivity of 20–30%, although the specificity was in the region of 90% for pancreatic endocrine tumours [6]. The main reason that contrast enhanced CT fails to detect insulinoma is that the tumour remains iso-attenuating to the pancreatic parenchyma. In a retrospective study with 181 insulinoma patients, 24.9% of the tumours were iso-attenuating on biphasic CT [7]. In a prospective comparison of CT (biphasic and volume perfusion) and 3-Tesla MRI with diffusion weighted imaging for insulinoma detection, MRI demonstrated a superior sensitivity of 90% with an equal specificity of 76.5%. Furthermore, it had higher tumour conspicuity and superiority in depicting tumour to duct distance [8].

PET with novel tracers such as 5-Hydroxytryptophan has shown to be promising in localizing gastrointestinal neuroendocrine tumours [9]. In a prospective study of 10 patients with confirmed hyperinsulinaemic hypoglycaemia and presumed insulinoma, PET scan with 5-Hydroxytryptophan was able to localize the pancreatic lesions in 9 patients which was confirmed by histological analysis [10]. But PET with 18F-flourodeoxyglucose (FDG) as the prime tracer was unable to identify well differentiate and slow growing pancreatic tumours [11]. Our patient also had a well differentiated pancreatic islet cell tumour, which could be the probable cause for the negative PET imaging. Conventional PET imaging was only able to localize less differentiated gastroentero-pancreatic tumours with high proliferative activity [12].

In patients with hyperinsulinaemic hypoglycaemia with negative noninvasive imaging, invasive tests such as SACST and endoscopic ultrasonography are useful in localization of insulinoma. SACST is a dynamic test based upon the observation that calcium stimulates the release of insulin from hyperfunctioning beta cells, but not from normal beta cells. Selective infusion of calcium gluconate into gastroduodenal, splenic and superior mesenteric arteries followed by effluent venous sampling for insulin allows both diagnosis and localization of insulinoma to respective arterial territory. The sensitivity of SACST for localization of insulinoma was noted to be significantly higher than noninvasive imaging modalities [5, 13–15]. Its value in localizing occult insulinomas is well established [15–19]. However, due to its invasive nature, SACST should be considered as the last resort in the evaluation of insulinomas [17].

The current treatment modality of choice for insulinoma is surgical resection. The procedure selected may vary from enucleation of the tumour, partial or complete pancreatectomy to Whipple’s procedure, depending on patient and tumour characteristics and availability of resources. In the Mayo clinic cohort, surgical resection was curative in about 87.5%. Major complications were observed in 10% of patients. It was also observed that the risk of recurrence of insulinoma was significantly higher among patients with MEN 1 (21% vs. 5% at 10 years) syndrome than in those without MEN 1 syndrome [20]. A significant proportion of MEN 1 syndrome patients were found to have multiple insulinomas and required repeated surgical interventions [21–23]. Hence it was recommended that patients with insulinoma related to MEN 1 syndrome need to undergo local excision of the tumour combined with distal subtotal pancreatectomy. But some studies concluded that enucleation and local resection provided long term cure for MEN 1 syndrome associated insulinoma in patients with solitary or dominant tumours while subtotal distal pancreatectomy should be preserved for patients with multiple insulinoma without dominance [24]. This approach is suggested to minimize the risk of exocrine and endocrine pancreatic insufficiency in a relatively young population.

Chemoembolization as an alternative treatment has been evaluated in patients with insulinoma. It was noted to have successful outcomes in a few case reports [25–27]. However, further evidence is necessary to establish this as a definitive curative treatment modality.In our patient, transarterial chemoembolization was attempted prior to surgery which was unsuccessful and precipitated severe acute pancreatitis with multi-organ failure.

Cutaneous tumours such as multiple angiofibromas, collagenomas, lipoma, hyperpigmented macules and multiple gingival papules are frequently associated with MEN 1 syndrome [28, 29]. Among these lesions, angiofibromas and collagenomas are noted be more frequent (77–81%) and multiple in number in patients with MEN 1 syndrome [30]. A prospective study designed to evaluate the significance of cutaneous lesions in diagnosis of MEN 1 syndrome concluded that, angiofibromas or collagenomas (single or multiple) had clinically significant diagnostic sensitivity of 50–65% and a specificity of 92–100%. A combination criterion of 3 or more angiofibromas and any number of collagenomas had higher sensitivity and specificity for diagnosis of MEN 1 syndrome [30]. The criterion was comparable in sensitivity to detecting hyperparathyroidism at the presentation of gastrinoma in some studies [30, 31]. It had superior sensitivity when compared to presence of pituitary disease and adrenal tumours in MEN 1 [31–33]. The physicians should be aware of the clinical significance of these seemingly benign lesions that may point to a more sinister underlying pathology. These lesions should be actively sought for during clinical examination of MEN 1 syndrome suspected patients and screening of their first-degree relatives to aid in a presymptomatic diagnosis [28]. But it should be also remembered that these cutaneous lesions are not pathognomonic for MEN 1 syndrome and are seen to occur in association with other clinical syndromes such as Tuberous sclerosis and Burt-Hogg-Dube syndrome.

In a young patient with primary hyperparathyroidism complicated with nephrolithiasis and family history of nephrolithiasis, possibility of MEN 1 syndrome should always be considered [34, 35]. Patients with MEN 1 syndrome related primary hyperparathyroidism (PHPT) exhibit higher susceptibility to nephrolithiasis than non-MEN 1 syndrome patients [34, 36, 37]. There is a higher frequency of renal calculi in MEN 1 syndrome patients before 30 years of age [34].

MEN 1 syndrome occurs in both familial and sporadic forms and differentiating between two forms is not easy. Among the two forms, familial form is frequently observed (90%) [38]. Absence of family history is observed even among some cases of familial form which may be attributed to non-paternity, early parental death, lack of careful family assessment and adoption [39]. Hence there is a possibility of familial cases getting falsely labeled as sporadic cases. An in-depth assessment of family history is imperative to avoid such clinical errors.

Patients with MEN 1 syndrome appear to have a significantly low survival compared to average population [20, 24]. In a retrospective study among a cohort of MEN 1 syndrome, 28% of patients died of causes related MEN 1 syndrome, most commonly due metastatic islet tumours. In another study, 70% of patients with MEN 1 syndrome died of causes related MEN 1 syndrome [40, 41]. Due to significant morbidity and mortality related to MEN 1 syndrome, it is suggested that affected patients should undergo annual surveillance for pancreatic neuroendocrine tumours using plasma hormonal measurements and imaging studies [2]. This may permit timely diagnosis and interventions which in turn will minimize debilitating complications such as hypoglycaemia induced brain injury.

In conclusion, renal calculi or characteristic cutaneous lesions might be the only forewarning clinical manifestations of an undiagnosed MEN 1 syndrome, impending a life-threatening presentation such as hypoglycaemic encephalopathy. Clinicians should be highly vigilant and should actively look for other associated clinical signs and family history in such patients. If in doubt,it is advisable to arrange periodic reviews to detect any new clinical symptoms and signs to minimize clinical error. Comprehensive management of MEN 1 syndrome requires multi-disciplinary approach with advanced imaging modalities, advanced surgical procedures and long term follow up.

## Data Availability

All necessary data and material are provided.

## Abbreviations

MEN 1: Multiple endocrine neoplasia type 1
PTH: Parathyroid hormone
TSH: Thyroid stimulating hormone
fT4: Thyroxin
FSH: Follicular stimulating hormone
LH: Luteinizing hormone
CECT: Contrast Enhanced Computed Tomography
MRI: Magnetic resonance imaging
FLAIR: Fluid-attenuated inversion recovery
PET/CT: Positron Emission Tomography—Computed Tomography
MDT: Multidisciplinary team
SACST: Selective arterial calcium stimulation test
FDG: Fluorodeoxyglucose
